# Environmental Transformational Leadership and Green Innovation in the Hotel Industry: Two Moderated Mediation Analyses

**DOI:** 10.3390/ijerph192416800

**Published:** 2022-12-14

**Authors:** Ibrahim A. Elshaer, Manal A. Abdelrahman, Alaa M. S. Azazz, Mahmaod Alrawad, Sameh Fayyad

**Affiliations:** 1 Department of Management, College of Business Administration, King Faisal University, Al-Ahsaa 380, Saudi Arabia; 2 Hotel Studies Department, Faculty of Tourism and Hotels, Suez Canal University, Ismailia 41522, Egypt; 3 Quantitative Method Department, College of Business Administration, King Faisal University, Al-Ahsaa 380, Saudi Arabia; 4 Department of Tourism and Hospitality, Arts College, King Faisal University, Al-Ahsaa 380, Saudi Arabia; 5 Tourism Studies Department, Faculty of Tourism and Hotels, Suez Canal University, Ismailia 41522, Egypt; 6 College of Business Administration and Economics, Al-Hussein Bin Talal University, Ma’an 71111, Jordan

**Keywords:** hotels industry, environmental transformational leadership, organizational citizenship behaviors for the environment, green innovation, green perceived organizational support, promotion focus

## Abstract

This research aims to investigate the relationships between transformational environmental leadership (ETL), organizational citizenship behavior for the environment (OCBE), and green innovation (GI). At the same time, green perceived organizational support (GPOS) and promotion focus (PF) roles were examined as moderators. Integrating transformational leadership, ability-motivation-opportunity (AMO), social exchange, expectancy, and regulatory focus theories, a quantitative research-based methodology was adopted with structural equation modelling (SEM), and smart partial least squares (PLS) program to analyze the obtained data from 388 guest-contact employees. The results show that ETL positively affects OCBE; in return, OCBE mediated the association between ETL and GI. The results also approved the positive moderation effects of the GPOS and PF variables on the association between ETL and OCBE. In the context of the hotel industry, we discuss the implications that these results have for both research and practice.

## 1. Introduction

Recent research has shown that corporations are to blame for climate change because they do not stop emitting harmful chemicals and carbon dioxide into the air and water [1]. One of the sectors that is extremely susceptible to the impending threat posed by climate change is the tourism industry, particularly the hotel sector. The hotel industry’s high energy and water use, paper, plastics, and chemical use, and biodiversity impact contribute significantly to global warming [2,3]. Therefore, hotels are obligated to implement environmental and green practices. Indeed, the hotel industry pioneered environmentally friendly methods in the tourism industry. Adopting green practices in hotels has become a key management strategy [4,5]. This is primarily due to managers’ awareness of the importance of these practices [4].

Previous research indicates that employees across organizational functions and levels significantly impact environmental performance [6], so the application of strategic environmental procedures relies on supervisory leaders [7]. According to transformational leadership theory, transformational leaders are considered to be more efficacious in improving environmental performance [8]. Environmental transformational leadership (ETL) could be the best predictor to enhance green innovation and organizational citizenship behaviors for the environment (OCBE) [9]. 

According to the theory of ability, motivation, and opportunity (AMO), ETL can help hotels practice green innovation for outstanding environmental performance [10]. According to the theory of social exchange, ETL can also boost a high-quality reciprocal association with subordinates. Therefore, employees voluntarily step up to take on additional responsibilities in order to implement positive behaviors such as OCBE [11]. In the meantime, hotel workers with a high OCBE can prepare to investigate and propose novel environmentally friendly ways to reduce environmental damage [11]. In the meantime, hotel workers with a high OCBE can prepare to investigate and propose novel environmentally friendly ways to reduce environmental damage [11].

Based on expectancy theory, green perceived organizational support (GPOS) could boost the role of ETL in increasing OCBE behaviors by motivating the employees to perform more green discretionary behaviors [12]. Similarly, the regulatory focus theory (RFT) would suggest that employees with a high promotion focus (PF) could support the role of OCBE in boosting green innovation [13,14].

Previous research primarily focused on reaching green targets via adopting government procedures and green technology innovation, and most studies have failed to recognize the importance of the environmental behaviors of their employees [15]. Although ETL is a critical organizational component, individual antecedents such as OCBEs are essential but missing in the existing literature, such as [16,17,18,19,20]. Therefore, some previous studies recommend additional research on mediating factors between transformational leadership and innovations [21,22,23]. To bridge this gap, the current research seeks to combine these two axes by relying on the transformational leadership theory, the ability, motivation, and opportunity (AMO) theory, the “social exchange theory”, the expectancy theory, and the regulatory focus theory (RFT) to examine the direct relationship between transformational environmental leadership (ETL) and green innovation (GI), with the mediating effect of the OCBE and the moderating effects of green perceived organizational support (GPOS) and promotion focus (PF) between OCBE and green innovation (GI) and between transformational environmental leadership (ELT) and OCBE, respectively.

## 2. Theoretical Context and Hypotheses Formation

### 2.1. Environmental Transformational Leadership (ETL)

Transformational leaders in organizations are viewed to be more efficient in boosting overall environmental performance [8]. In 2013, Robertson and Barling introduced the transformational environmental leadership (ETL) concept based on the transformational leadership theory. They defined it as “a manifestation of transformational leadership in which content of the leadership is all focused to encourage pro-environmental and green initiatives.” Transformational leadership was categorized into four dimensions, “charisma or idealized influence, inspirational motivation, individualized consideration, and intellectual stimulation” Bass [24]. These behaviors could be used to influence the environmentally friendly actions of employees. Charisma or idealized influence indicates that the transformational leader serves as an example for his followers. Thus, the followers will adopt green values and convictions when their transformational leaders adopt them. Using inspirational motivation ability, transformational leaders can inspire and motivate their subordinates to engage in environmentally conscious actions. Through green intellectual stimulation capability, leaders inspire their followers to challenge presumptions and solicit ideas to overpower environmental issues with innovative approaches. Lastly, via individualized consideration, transformational leaders use attention to the individual concerns of their subordinates to create a mutually strong relationship to transfer their pro-environmental values to them [7,25,26]. Simply put, transformational environmental leaders encourage and inspire their followers to employ pro-environmental behavior through the use of their idealized charismatic personalities.

### 2.2. OCBE as a Mediator

OCBE has garnered the interest of numerous academics [15] since it was first offered by Boiral [27]. OCBE is defined based on the same principles as OCB as “individual and discretionary social behaviors not explicitly acknowledged by the formal reward system and contributing to improve the effectiveness of environmental management of associations” [27]. There is agreement among many studies that OCBE can improve environmental performance [28]. In addition to filling gaps in the formal environmental management system (EMS), this initiative will enable the association to reduce environmental costs and enhance its ecological reputation [15].

OCBE behaviors consist of three main categories on which several studies have depended. The first one is eco-helping, which can be defined as assisting one another regarding environmental concerns, such as sharing green knowledge and helping coworkers to engage in more environmentally friendly behavior; the second one is eco-civic engagement, which motivates employees to willingly participate in environmental events and affairs regarding the organization’s ecological issues; and the third is eco-initiatives, including workplace environmental initiatives (recycling, reducing water and energy consumption, etc.), pro-environmental suggestions, volunteer initiatives aimed at reducing greenhouse gas emissions, etc., suggested by employees [29,30,31]

Transformational leadership can improve the quality of a reciprocal relationship by expressing care, confidence, and support directly to followers [15]. By directly expressing care, confidence, and support to followers, transformational leadership can foster a high-quality reciprocal relationship [32]. Therefore, according to the social exchange theory, ETL may effectively motivate and stimulate subordinates to display OCBE behaviors [11]. Consequently, we can hypothesize the following:

**Hypothesis** **1** **(H1).** *ETL are positively related to OCBE*.

Innovation is the organizational capacity to adopt and successfully implement novel ideas, products, and procedures [33], whereas green innovation (GI) reflects the steps taken to reduce the potentially harmful consequences that operations and production might affect the environment, with a focus on enhancing the procedures, technologies, systems, and products, as well as management techniques [34,35]. GI adopts creativity practices, namely greener raw materials, utilizing fewer materials during the product design, employing design measures, and seeking to reduce emissions, water, electricity, and other natural materials’ consumption [36].

Hotels’ success primarily depends on introducing innovative services to gain a larger market share [37,38]. Since customers are becoming more environmentally conscious and concerned, hotels have begun to demonstrate their commitment to developing eco-friendly techniques, such as green innovation, as an effective means of combating market competition [39]. Green innovation is an inevitable way to re-engineer or remanufacture products and services to become more environmentally friendly [40].

Research reveals that OCB is positively linked to innovative behavior since both usually need extra work out of employees’ obligations [41] Some studies also proved a positive relationship between OCB and individual creativity [42]. In the hotel sector, creativity is a crucial footstep in the innovation process and an initial point for organizational innovation [43]. Thus, according to [11], hotel employees with high OCBE would demonstrate more green innovation activities that help explore and offer innovative ways to decrease environmental damage. Based on these arguments, the following is hypothesized:

**Hypothesis** **2** **(H2).** *OCBE is positively related to GI*.

To succeed in developing innovations, organizations must first cultivate an innovation-friendly and adaptable culture. In line with this, transformational leaders drive innovation within the organization through inspirational motivation, intellectual stimulation, and supporting freedom to subordinates to determine what they wish to work on and how to achieve their objectives [23,44]. Consequently, prior research indicates that transformational leadership is crucial to organizational innovation [44,45] through OCBE [32]. Based on these arguments and consistent with the AMO theory, the following hypothesis is proposed:

**Hypothesis** **3** **(H3).** *OCBE mediates the link between ETL and GI*.

### 2.3. GPOS as a Moderator on ETL and OCBE

The GPOS reflects the beliefs and perceptions of staff members about how their company appreciates their environmentally friendly contributions and practices [46,47]. GPOS has received growing attention from several researchers due to its direct effect on employees’ green behaviors [48]. Previous research has demonstrated a positive connection between GPOS and subordinates’ attitudes and behaviors, including job satisfaction, OCBE, and green creativity [48,49]. The GPOS also increases the employees’ belonging to the work’s social network, thus improving their acceptance of organizational values and norms and their compatibility with them [50]. According to the expectancy theory, the GPOS guarantees that subordinates who have been treated relatively fairly receive a reward, such as recognition from the leaders, if they meet the performance expectations on demonstrating discretionary work [12,51]. According to social exchange theory, when employees feel green-supported, they will behave environmentally friendly way and enact volunteer environmental practices (OCBE) [52]. Accordingly, this study suggests the following hypothesis:

**Hypothesis** **4** **(H4).** *GPOS moderate the influence of ETL on OCBE (such that the relationship will be stronger when GPOS are high)*.

### 2.4. PF as a Moderator on OCBE and GI

**Figure 1:**
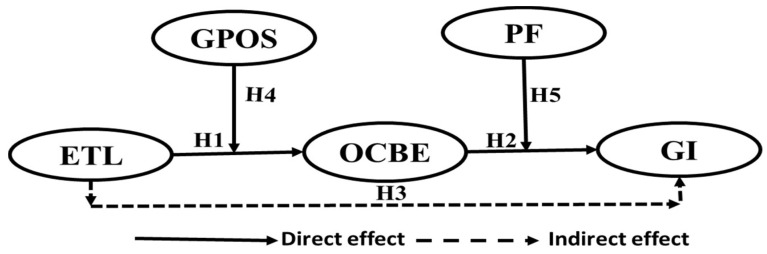
The developed framework with hypotheses. ETL → ”Environmental Transformational Leadership”; OCBE → ”Organizational Citizenship Behavior for the Environment”; GI → “Green Innovation”; GPOS → “Green Perceived Organizational Support”; and PF → “Promotion Focus”.

According to regulatory focus theory, employees adopt various reaching or task engagement tactics to achieve desired outcomes. This theory has two components: an emphasis on promotion and a separate emphasis on prevention [53]. Employees use a promotion focus strategy to approach improvement, aspirations, growth, and accomplishments, and they constantly strive to achieve positive results and avoid non-gain situations [53,54]. Otherwise, prevention-focused employees always pursue maintenance and safety, sticking to their obligations and following “ought to do” responsibilities to secure stability. They believe “avoidance” is a suitable strategic approach [55,56,57]. Regulatory focus theory hypothesizes that promotion focus is associated with innovative performance. This is because people with a promotion focus are more willing to tolerate ambiguity and take risks to provide creative ideas and solutions, in contrast to employees with a prevention focus [13,14,57]. Thus, promotion-focused employees do not just do OCBE behaviors but innovate in OCBE behaviors. This leads us to propose the below hypothesis, as in Figure 1:

**Hypothesis** **5** **(H5).** *PF moderates the influence of OCBE on GI, such that the relationship will be stronger when PF are high*.

## 3. Materials and Methods

### 3.1. Measurement Development

A questionnaire was developed to test the study’s hypotheses, and the measures were extracted from a comprehensive, in-depth analysis of prior empirical research. Five dimensions have materialized as a direct consequence of the process that came before. According to the findings of the study, the ETL was evaluated using six different factors derived from Crowe and Higgins [58]. The OCBE was operationalized using the seven-item scale suggested by Boiral and Paillé [29]. Four items from Asadi et al. [35] were used to operationalize GI. The GPOS was evaluated using the six-items scale proposed by Paillé and Meija-Morelos [52]. Finally, six items from Wallace and Chen [59] were used to measure PF. A Likert scale of five points was used, where one refers to “strongly disagree” and five means “Strongly agree”. Some academics and professionals validated the scale with no significant modifications.

### 3.2. Participants and Process of Data Collection

Three of the five research teams were previously employed in various tourism and hotel management faculties. Therefore, they used their personal network and connections with hotel managers to disseminate the developed survey. Questionnaires were directed to first-line line employees who had direct contact with guests at Sharm El-Sheikh hotels (Egypt) during June 2022, using convenient sample and drop-and-collect methods. The research team handed out a total of 500 questionnaires. The city of Sharm El-Sheikh was chosen because it has a great number of hotels that are rated five stars or higher. Only employees with at least three years of experience were allowed to take the survey. Of the 500 questionnaires, 112 were discarded because they lacked sufficient responses. This resulted in a recovery rate of 77.6%, as 388 questionnaires were found to be valid. Respondents were required to sign a consent form and could either take part in the survey or skip it. All respondents were assured that the results of their participation in the survey would be kept private. Participants in the study’s sample ranged in age from 24 to 57 years old, with 67.8% being male and 32.2% being female. 80.4% of the respondents hold a bachelor’s degree, which is an overwhelming majority. A little more than half of the participants, or 58.2%, have more than five years of experience working in hotels, while the remaining participants, or 41.8%, have between three and six years of experience. The percentage of workers who were single came in at 30.2%, which was lower than the number of married workers (69.8%).

An independent *t*-test sample method was utilized in order to carry out an investigation into possible non-response bias. As a result of the fact that the mean-variance between late and early responses did not display any significant statistical value (*p* > 0.05), bias from non-response is not a concern in this study [53].

## 4. Results of Data Analysis

This research aims to give answers that explain and test the impact of transformational environmental leadership on green innovation through the mediating role of organizational citizenship behavior for the environment and the moderating role of green perceived organizational support and promotion focus. A quantitative-based research methodology was adopted to achieve the research aims by employing a fully structured online survey to gather the required data. Partial least squares-based structural equation modelling (PLS-SEM) was employed as the primary data analysis technique. PLS-SEM is a suitable method for testing and validating the early phases of theory improvement [60]. PLS-SEM was used to examine the measurement and structural models due to its multivariate and predictive benefits with a small sample. Furthermore, 5000 bootstraps repeated 388 samples to assess the significance of the path coefficient for a more accurate determination of coefficient values. The model was evaluated using Leguina’s [60] two-step sequential technique.

### 4.1. Outer Model Evaluation

We looked at discriminant and convergent validity, internal consistency, and composite reliability to evaluate the outer model. Cronbach’s alpha (α) and composite reliability (CR) are presented in Table 1; they range from 0.886 to 0.943 and 0.914 to 0.954, respectively, which indicate proper reliability.

Second, all standardized factor loading (SFL) scores were greater than 0.60 [53], indicating that the factors had satisfactory reliability. The average variance extracted (AVE) scores were greater than the threshold value of 0.50 in a third, evidence for a proper convergent validity [53]. Finally, three criteria were checked to test the discriminant validity: heterotrait–monotrait ratio (HTMT), cross-loading, and Fornell–Larcker criterion [60]. Outer-factor loading for each latent observed variable (bolded) was greater than cross-loading in Table 2.

Table 3 demonstrates that the bolded scores of the squared AVEs on the diagonal line exceed the correlation coefficient between the research variables, which supports discriminant validity [54]. Furthermore, all HTMT values were found to be less than 0.90, which gives more signals to support discriminant validity [51]. Together, the results demonstrated that the structure model has sufficient discriminant validity. In this way, the results from the outer measurement model were adequate to move forward with the structural model evaluation.

### 4.2. Inner Model Evaluation

After testing and guaranteeing that the employed scale has adequate convergent and discriminant validity, the inner structure mode was evaluated regarding the structure inner model’s predictive and explanatory power [61]. The VIF values for all the observed variables vary from 1.657 to 4.052. These numbers are lower than the recommended threshold of 5.0, which shows that there is no multicollinearity in the structural inner model. Chin [56] suggested a minimum R2 value of 0.10 for adequate GoF. As shown in Table 4, the R^2^ values for the OCBE (R^2^ = 0.510) and GI (R^2^ = 0.366) are adequate. Additionally, the Stone–Geisser Q^2^ evaluation demonstrated that the OCBE and GI values were higher than zero (Table 4), indicating a proper predictive power of the structural inner odel [57].

**Figure 2:**
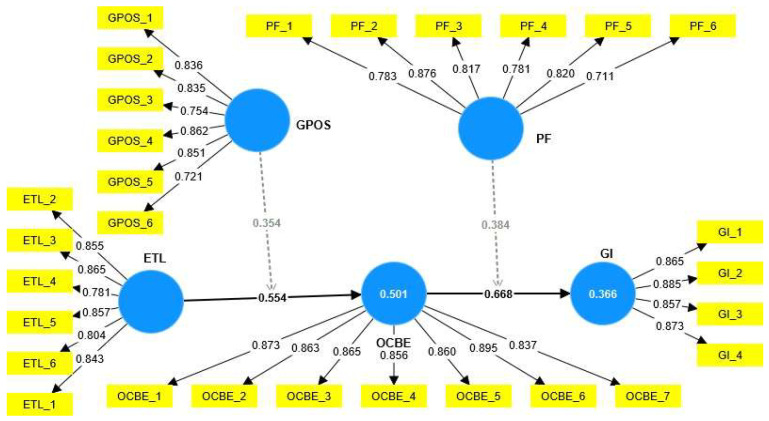
The inner and outer model results. ETL → ”Environmental Transformational Leadership”; OCBE → ”Organizational Citizenship Behavior for the Environment”; GI → “Green Innovation”; GPOS → “Green Perceived Organizational Support”; and PF → “Promotion Focus”.

Finally, the direct and specific indirect effects were examined (Table 5 and Figure 2) using the bootstrapping option in the SmartPLS program to evaluate the study hypotheses. All direct, mediating, and moderating assumptions were assessed through the path coefficient (**β**), significance *p*-values, and the related t-value. ETL has a significant positive association with OCBE at β = 0.554, with *p* < 0.001; thus, H1 is accepted. OCBE was found to have a significant positive relationship with GI (β = 0.668, with *p* < 0.001), we then can support H2. As for the effects of mediation, ETL was found to positively impact GI through OCBE at β = 0.370, with *p* < 0.01, supporting H3. Finally, the results approved the moderation effects of GPOS and PF on ETL and GI at β = 0.354, with *p* < 0.01, β = 0.384, with *p* < 0.01, respectively, supporting H4 and H5.

## 5. Discussion and Implications

### 5.1. ETL, OCBE, and GI

The SEM results indicated that the ETL has a positive effect on OCBE. This finding agrees with the AMO theory principles, whereby the transformational leaders’ practices in an organization aim to attract, motivate, reward, and sustain employee job behaviors toward achieving goals and objectives in environmental management through boosting OCBE behaviors and green innovation for superior green performance [23,62]. In addition, according to the social exchange theory, ETL may effectively drive employees to enact OCBE behaviors [11]. The results also showed that the OCBE positively affects GI. In line with this finding, Naqshbandi et al. [63] indicate that OCBs, in general, are one of the most vital unstudied factors that can play a crucial role in the success of the innovation process in organizations. OCB facilitates the innovation process via cooperative and collaborative actions that do not need formal organizational boundaries [64]. As for the OCBE, Öğretmenoğlu et al. [11] argue that hotel employees with high OCBEs would engage in more green innovation activities that enable exploration and offer innovative ways to reduce environmental damage.

### 5.2. Evaluating the Moderating Effect

**Figure 3:**
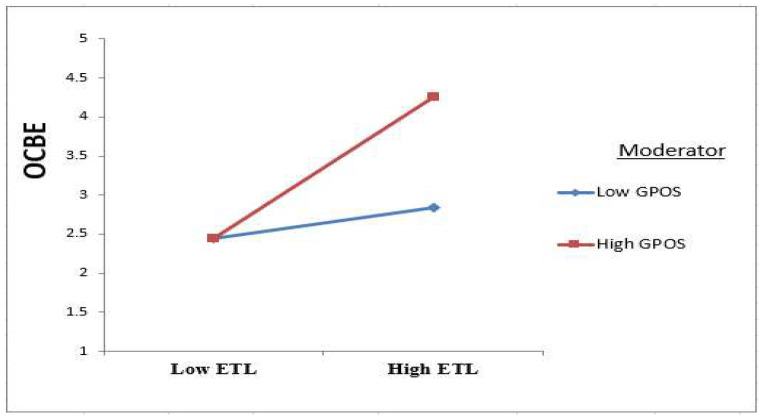
Plot for the GPOS as a moderator on ETL towards OCBE.

The empirical results validated the positive moderation effects of the GPOS variable on the relationship between ETL and OCBE. In other words, according to the interaction plot in Figure 3, GPOS made the connection between ETL and OCBE will strengthen. This result agrees with the findings of Paillé and Meija-Morelos [52], who argue that employees, according to social exchange theory, when feeling green supported, will behave in a green way and enact discretionary environmental practices (OCBEs). Indeed, these GPOSs make the employee feel psychological ownership of the hotel, and the feeling that a thing is “mine” enhances attitudes toward it and attachments to it, and boosts its perceived value [65]. According to the expectancy theory also, employees consider POS a reward from the leaders because they do an extra-role out of their obligations [12,51]. Thus, we argue that GPOS is a guarantee of the success of ETL in encouraging the employee to demonstrate OCBEs.

**Figure 4:**
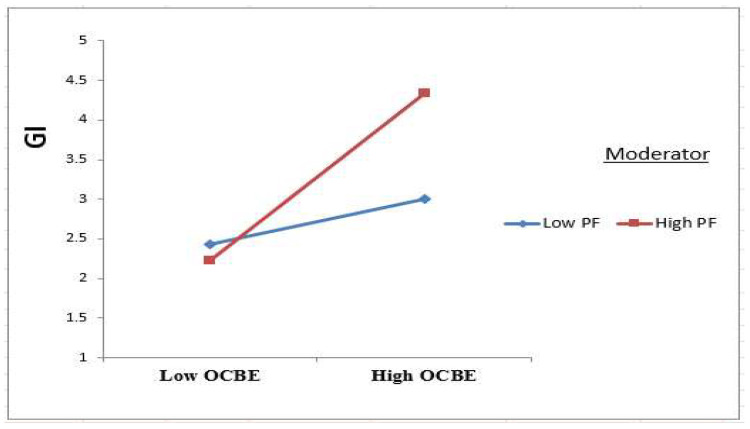
Plot for the PF moderation impact on OCBE towards GI.

On the other hand, the practical results also validated the positive moderation influences of the PF variable on the relationship between OCBE and GI. According to the interaction plot in Figure 4, PF means the connection between OCBE and GI will strengthen. This result agrees with the regulatory focus theory that asserts that promotion-focused employees are ready to accept tolerance for ambiguity and risk-taking to provide creative ideas and solutions, so they are related to innovative performance in contrast to prevention-focus employees [13,14,57]. Since promotion-focused employees strive for growth and development and seek ideals, aspirations, and rewards via accomplishment, they use innovative ways to use OCBE to improve hotel environmental performance [66,67].

### 5.3. The Mediating Role of OCBE between the Relationship ETL and GI

One of the study’s primary objectives was to examine the mediating function of OCBE between ETL and GI. The study’s results showed that OCBE has positively and significantly mediated the association between ETL and GI. This result is consistent with the investigations of Singh et al. [23] and Gumusluoglu and Ilsev [44] indicate that transformational leaders motivate employees to enact OCBEs through intellectual stimulation, inspirational motivation, and providing freedom to employees to decide what they want to work on and how to reach their goals. This organizational climate is facilitative to the successful development of innovations. In brief, ETL plays a crucial role in GI through OCBE.

## 6. Conclusions

According to the theory of transformational leadership, transformational leaders are more effective at enhancing environmental performance. Environmental transformational leadership may be the most accurate predictor of green innovation and organizational environmental citizenship behavior. Using the AMO theory, transformational environmental leadership can encourage hotels to engage in green innovation for exceptional environmental performance. Moreover, according to the social exchange theory, transformational environmental leadership can foster a high-quality reciprocal relationship with subordinates. Thus, employees volunteer to implement positive behaviors, such as organizational citizenship behavior for the environment (OCBE), as part of their responsibilities. Meanwhile, hotel employees with high OCBE can be ready to explore and propose innovative green ways to reduce environmental damage. Our study further tests the moderating effects of green perceived organizational support (GPOS) and promotion focus (PF) in the tested relationships based on expectancy theory and regulatory focus theory (RFT).

Data were collected from 388 first-line employees at Sharm El-Sheikh hotels (Egypt). Convergent and discriminant validity and the research hypotheses were evaluated by conducting SEM with the Smart PLS program. The findings approved that the scale has good validity. Furthermore, the findings showed that ETL positively affects OCBE; in return, OCBE has thoroughly and significantly mediated the association between ETL and GI. The results also validated the positive moderation effects of the GPOS and PF variables on the relationship between ETL and OCBE.

This research has several theoretical and practical implications. In terms of theoretical implications, the current study used the transformational leadership theory to prove that ETL practices enhance employees’ abilities, motivations, and opportunities to contribute, based on the AMO theory, to OCBEs and GI. Correspondingly, according to the social exchange theory and the expectancy theory, our study used the GPOS as a managerial and leadership tool to support OCBEs, and used PF as an individual self-regulation tool (personal tool) to improve the effect of OCBE on PF. Thus, the study adds to the knowledge related to green behavior literature. Furthermore, the literature on ETL and its antecedents and outcomes is still emerging, and its influence on subordinates’ outcomes is still in its infancy [7]. Regarding practical implications, this paper strives to help fill a gap of a lack of agreement on the effective mechanisms that can influence employees’ green behavior [68]. Therefore, the authors examined the role of OCBE in mediating the relationship between ETL and GI. At the same time, GPOS and PF were tested as moderators between ETL and OCBE and OCBE and GI, respectively. Furthermore, the study recommends hotels and their management support the employee through GPOS to cope with the workplace environment through task-coping styles [68] to boost ETL practices to instill and stimulate OCBE behaviors and support the employees’ self-regulation method to be PF, which helps develop green innovations. Finally, the study provides implications for policymakers and practitioners in the hotel industry that an environmental transformational leadership style can not only improve the image of the company but also can generate environmentally friendly innovative performance and pave the way to build a strong competitive advantage. This can happen with the support of top management and designing promotion policies that reinforce and foster green practices.

Similar to previous research on this topic, the current study has several limitations, and it is suggested that additional research avenues can be explored. First, the study investigated the impact of transformational environmental leadership (ETL) and green innovation (GI) on organizational citizenship behavior for the environment (OCBE), which was employed as a mediating variable, while green perceived organizational support (GPOS) and promotion focus (PF) roles were examined as moderators; however, other variables can be investigated as mediating, such as supervisors–subordinate trust, and distributive justice, while different factors can be tested as mediators, such as job satisfaction or years of experience. Second, cross-sectional data precludes precise causal effects between latent variables. Future researchers may employ longitudinal or multiple data sources to verify the study’s structure model. Finally, a method of multi-group analysis can evaluate these relationships in a context that is distinct from our contexts, such as different countries or industries.

## Figures and Tables

**Table 1:** Outer model evaluation statistics.

Variables		Loading	Mean	S.d	α	C_R	AVE
ETL			3.56	0.85	0.914	0.932	0.697
ETL1	The leader inspires the organization members with the environmental plans.	0.843					
ETL2	The leader provides a clear environmental vision for the members to follow.	0.855					
ETL3	The leader gets the organization members to work together for the same environmental goals.	0.865					
ETL4	The leader encourages the organization members to achieve the environmental goals.	0.781					
ETL5	The leader acts with considering environmental beliefs of the organization members.	0.857					
ETL6	The leader stimulates the organization members to think about green ideas.	0.804					
OCBE			3.52	1.13	0.943	0.954	0.747
OCBE1	I suggest new practices that could improve the environmental performance of my organization	0.873					
OCBE2	I encourage my colleagues to adopt more environmentally conscious behavior	0.863					
OCBE3	I stay informed of my organization’s environmental initiatives	0.865					
OCBE4	I make suggestions about ways to protect the environment more effectively	0.856					
OCBE5	I volunteer for projects or activities that address environmental issues in my organization	0.860					
OCBE6	I spontaneously give my time to help my colleagues take the environment into account	0.895					
OCBE7	I undertake environmental actions that contribute positively to the image of my organization	0.837					
GI			3.82	0.96	0.893	0.926	0.757
GI1	Our hotel industry uses less or non-polluting/toxic materials.	0.865					
GI2	Our hotel industry improves environmentally friendly packaging for existing and new products.	0.885					
GI3	Our hotel industry recovers of hotel’s end-of-life products and recycling.	0.857					
GI4	Our hotel industry uses eco-labeling.	0.873					
GPOS			3.91	0.77	0.900	0.920	0.658
GPOS1	The organization takes pride in my environmental accomplishments at work	0.836					
GPOS2	My colleague really cares about my view on the environment	0.835					
GPOS3	The organization values my environmental contribution	0.754					
GPOS4	My organization is willing to assist employees in solving environmental problems	0.862					
GPOS5	My organization is willing to extend itself to solve an environmental problem	0.851					
GPOS6	Help is available in my company when environmental problems arise	0.721					
PF			3.76	0.76	0.886	0.914	0.639
PF1	I can always do a lot of work	0.783					
PF2	Anyway, I have to finish my work	0.876					
PF3	Can do a lot of work in a short time	0.817					
PF4	Work tasks can make me better at work	0.781					
PF5	I often wonder if my work is done	0.820					
PF6	I always think about how much work I can accomplish	0.711					

**Table 2:** Cross loading results.

Abbreviation	ETL	OCBE	GI	GPOS	PF
ETL_1	**0.843**	0.399	0.448	0.130	0.235
ETL_2	**0.855**	0.429	0.471	0.181	0.176
ETL_3	**0.865**	0.307	0.410	0.047	0.218
ETL_4	**0.781**	0.279	0.394	−0.005	0.249
ETL_5	**0.857**	0.336	0.366	0.030	0.243
ETL_6	**0.804**	0.445	0.420	0.005	0.277
OCBE_1	0.407	**0.873**	0.424	0.294	0.584
OCBE_2	0.381	**0.863**	0.409	0.278	0.492
OCBE_3	0.403	**0.865**	0.362	0.281	0.523
OCBE_4	0.384	**0.856**	0.414	0.199	0.572
OCBE_5	0.453	**0.860**	0.416	0.253	0.629
OCBE_6	0.357	**0.895**	0.449	0.199	0.679
OCBE_7	0.341	**0.837**	0.527	0.296	0.589
GI_1	0.446	0.466	**0.865**	0.116	0.425
GI_2	0.466	0.460	**0.885**	0.098	0.448
GI_3	0.479	0.375	**0.857**	0.095	0.424
GI_4	0.375	0.428	**0.873**	0.119	0.498
GPOS_1	0.022	0.310	0.148	**0.836**	0.161
GPOS_2	0.026	0.219	0.040	**0.835**	0.135
GPOS_3	0.099	0.057	0.038	**0.754**	−0.018
GPOS_4	0.056	0.243	0.093	**0.862**	0.031
GPOS_5	0.119	0.282	0.112	**0.851**	0.135
GPOS_6	0.138	0.181	0.111	**0.721**	0.098
PF_1	0.217	0.521	0.447	0.029	**0.783**
PF_2	0.228	0.564	0.454	−0.026	**0.876**
PF_3	0.153	0.496	0.366	0.067	**0.817**
PF_4	0.199	0.487	0.455	0.086	**0.781**
PF_5	0.304	0.610	0.399	0.260	**0.820**
PF_6	0.239	0.568	0.331	0.283	**0.711**

**Table 3:** Discriminant validiaty criteria.

		AVEs Values				HTMT Results				
	ETL	GI	GPOS	OCBE	PF	ETL	GI	GPOS	OCBE	PF
ETL	0.835 *									
GI	0.506	**0.870**				0.556				
GPOS	0.085	0.123	**0.811**			0.141	0.134			
OCBE	0.451	0.498	0.299	**0.864**		0.471	0.539	0.288		
PF	0.279	0.517	0.134	0.674	**0.799**	0.311	0.573	0.213	0.740	

* Bold values: squared AVEs.

**Table 4:** Model GoF.

Endogenous Latent Construct	(R^2^)	(Q^2^)
OCBE	0.501	0.348
GI	0.366	0.231

**Table 5:** The structural inner model’s findings.

	Hypotheses	Beta (β)	(T-Value)	*p* Values	Results
H1	ETL → OCBE	0.554	12.167	0.000	Supported
H2	OCBE → GI	0.668	6.891	0.000	Supported
H3	ETL → OCBE → GI	0.370	5.535	0.000	Supported
H4	GPOS × ETL → OCBE	0.354	10.917	0.000	Supported
H5	PF × OCBE → PF	0.384	7.164	0.000	Supported

## Data Availability

Data is available upon request from researchers who meet the eligibility criteria. Kindly contact the first author privately through e-mail.
